# PlasticHealthAware: Presenting the Latest Meta-Analytical Findings on Plastic Chemicals and Human Health

**DOI:** 10.5334/aogh.5195

**Published:** 2026-07-28

**Authors:** Yannick Mulders, Scott Cann, Christopher Carpenter, Thomas C. Chiles, Henoch Kabeya, Aoife Lyons, Saisha Shetty, Christopher Sonn, Christos Symeonides, Zahra Tahroudi, Kul Thapa, Philip J. Landrigan, Sarah Dunlop

**Affiliations:** 1 Minderoo Foundation, 171-173 Mounts Bay Road, Perth, WA 6000, Australia; 2 Boston College, 140 Commonwealth Avenue, Chestnut Hill, MA 02467, USA; 3 Centre for Community Child Health, Royal Children’s Hospital, Parkville, VIC, Australia; 4 Centre Scientifique de Monaco, Monaco, MC; 5 School of Biological Science, The University of Western Australia, 35 Stirling Highway, Crawley, WA 6009, Australia

Plastics are complex, manufactured chemical materials. Over 98% are produced from fossil fuels [1]. Across their life cycle, plastics pose substantial and increasing, yet insufficiently recognized, dangers to human and planetary health, contributing to disease, disability, and premature death from infancy to old age [2].

Plastics can contain more than 16,000 chemical additives [3, 4], either intentionally added into plastics or inadvertently incorporated during manufacture [5]. The Minderoo-Monaco Commission on Plastics and Human Health reported in 2023 that many of plastics’ harms to health are caused by these intentionally and unintentionally added chemicals, which can leach out of plastics, enter cells and tissues, and cause disease [6]. Primary research into plastics chemicals’ health hazards is increasing rapidly [7].

To develop a comprehensive overview of the evidence of plastics chemicals’ harms to health, Symeonides and colleagues undertook an umbrella review, identifying and extracting data from 52 reviews published through September 30, 2020 [8, 9]. The identified literature body focused on five widely used groups of plastic chemicals: bisphenols, brominated flame retardants (PBDEs), polychlorinated biphenyls (PCBs), per- and polyfluorinated substances (PFAS), and phthalates. Each chemical group was found to be significantly associated with at least one adverse human health impact, including reproductive, endocrine, neurodevelopmental, nutritional, circulatory, respiratory, dermatological, and cancer outcomes.

An unavoidable limitation of any systematic or umbrella review is that the information it contains begins to become outdated almost as soon as it is published. To overcome this problem of data obsolescence, the Global Observatory on Planetary Health at Boston College and the Minderoo Foundation have leveraged the already peer-reviewed and published methodology [8, 9] to create a pipeline that expedites the extraction and synthesis of newly reported data and regularly publishes this updated information in an interactive and approachable format. This produced the open-access dashboard: PlasticHealthAware (https://plastichealthaware.bc.edu/) [10], which was officially launched on August 9, 2025, in Geneva, Switzerland in a side event to the reconvened fifth session of the Intergovernmental Negotiating Committee (INC-5.2) for the Global Plastics Treaty [11].

We are now pleased to announce the first update to PlasticHealthAware [12], to include more recently published health outcome data and new features. The literature search and the screening and data extraction protocol [8, 9] were updated to include publications through August 2025. The search strategy focused on flame retardants, plasticizers, bisphenols, and PFAS:high-production-volume chemicals, chemicals present in high concentrations in plastics, and chemicals with known health concerns. Meta-analyses on exposures from medical interventions or those not measured *in vivo* in human biosamples were excluded. Two independent reviewers screened literature identified for inclusion in duplicate, and data from the included literature were subsequently extracted in duplicate again. Following a comprehensive quality control protocol, the new data were added to PlasticHealthAware. Supplementary documentation elaborating the methodology, search strategy, and extraction protocol is available on the dashboard.

**Figure 1:**
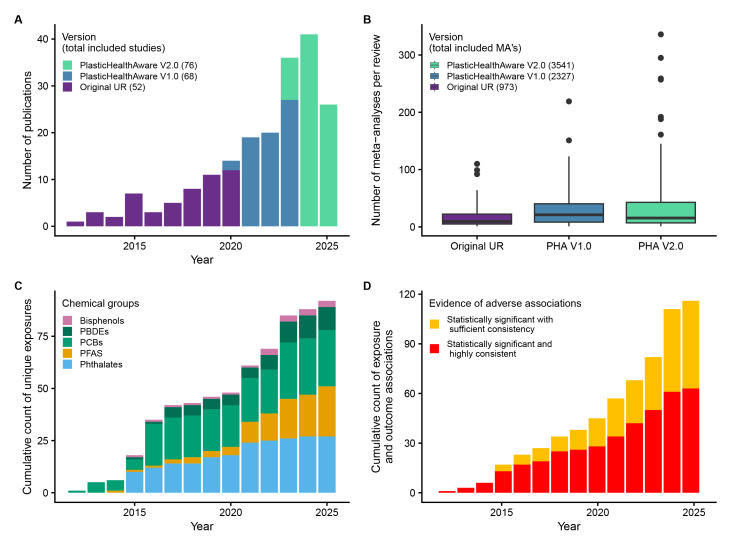
Number of reviews covering plastic chemical exposure and human health outcomes over time included in the original Umbrella Review (purple), the first version of PlasticHealthAware (blue), and the updated version of PlasticHealthAware (green) **(1A)**. Note that the search window was limited to August 2025. Distribution of included meta-analyses per review across the three versions **(1B)**. Boxes show the interquartile range (IQR) with the median as bold line, and whiskers extending to 1.5 x IQR beyond box edges. Cumulative number of unique plastic chemical exposures included in the reviews covered by PlasticHealthAware by publication year **(1C)**. Plastic chemical exposures are aggregated by group: bisphenols (pink), polybrominated diphenyl ethers (PBDEs - dark green), polychlorinated biphenyls (PCBs - light green), per- and polyfluoroalkyl substances (PFAS - yellow), and phthalates (blue). Cumulative number of adverse associations between plastic chemical group and health outcomes in the two strongest categories of evidence in PlasticHealthAware by publication year **(1D)**. Associations are considered “statistically significant and highly consistent” (red) when at least one meta-analysis finds a statistically significant adverse association, and all relevant meta-analysis findings are consistently in that same adverse direction. Associations are considered “statistically significant with sufficient consistency” (orange) when at least one meta-analysis finds a statistically significant adverse association, and with all relevant statistically significant findings in that same adverse direction, but with one or more contrary trends amongst findings that are not statistically significant.

With this update, PlasticHealthAware now presents data originating from 196 systematic reviews (Figure 1A), extracted from 6,841 meta-analyses (Figure 1B). On top of the yearly increase in the number of systematic reviews published, the number of meta-analyses within each review is also increasing. In the updated data, there were no new chemical groups included. However, the number of unique plastic chemical exposures studied within each group increased, with the largest increases seen for phthalates and PFAS (Figure 1C). Furthermore, additional significant adverse health outcomes were reported in association with plastic chemical exposures, including previously unrecognized associations (Figure 1D).

Rapid expansion of the body of evidence linking plastic chemicals to adverse health effects highlights the need for timely data synthesis and expanded access. Accordingly, we plan to update PlasticHealthAware at regular intervals to keep the findings current and improve user experience. Given the increasing publication rate in this field and the amount of novel chemicals being introduced to replace known chemicals of concern [13], we expect new plastic-associated exposures that fall within the current search strategy (e.g., organophosphate esters [OPEs] or micro- and nanoplastics [MNPs]) to be included in upcoming updates. Despite the use of a broader functional group approach (e.g., plasticizer and flame retardant), when applying the search strategy to the list of known plastic-associated chemicals [3], only a relatively small fraction (~20%) of these chemicals is covered. The establishment of the streamlined pipeline of extraction and synthesis will allow future updates to potentially apply a broader search strategy to also include other functional groups, such as UV stabilizers, pigments, and fillers.

PlasticHealthAware offers varying levels of detail for a range of stakeholders that include policymakers, intergovernmental organizations, NGOs, scientists, health practitioners, and the general public. Following a pyramid structure to guide the user journey, the dashboard engages users initially at a narrow scope, broadening as the user continues into the dashboard. At the highest level, adverse findings synthesized at chemical group level—currently bisphenols, PBDEs, PCBs, PFAS, and phthalates—are presented using human body outlines in a visual overview format (https://plastichealthaware.bc.edu/findings.php). Going deeper, a more orthogonal tabular format shows all levels of associations and also indicates potentially reassuring findings, as well as evidence gaps (https://plastichealthaware.bc.edu/evidence-grid.php). Finally, the data can be interrogated by health outcome, sex, and the stage of life at which a health effect occurs. In this last format, data can be downloaded and original sources accessed (https://plastichealthaware.bc.edu/figures-and-data.php). Suitable for both desktop and mobile devices, the dashboard uses a color vision deficiency safe palette and visual cues for as additional used guidance. To further increase accessibility, all information is made available in the six official UN languages: Arabic, Chinese, English, French, Russian, and Spanish.

Selective promotion or suppression of scientific findings by corporate and state entities to suit specific interests has led to the stagnation of the Global Plastics Treaty [14]. The inception of high-profile initiatives, such as the Lancet Countdown on Health and Plastics [2], highlights the need for transparent, independent, and accessible evidence that holistically evaluates the effects of plastics and plastic chemicals over the entire plastic life cycle. By keeping the data available on PlasticHealthAware up to date, we aim for it to become a relevant tool that can contribute to evidence-based policy on plastics at the global, national, regional, and local levels for the benefit of public health.
